# Core of the saliva microbiome: an analysis of the MG-RAST data

**DOI:** 10.1186/s12903-021-01719-5

**Published:** 2021-07-16

**Authors:** Simone G. Oliveira, Rafaela R. Nishiyama, Claudio A. C. Trigo, Ana Luiza Mattos-Guaraldi, Alberto M. R. Dávila, Rodrigo Jardim, Flavio H. B. Aguiar

**Affiliations:** 1 Department of Restorative Dentistry, Piracicaba Dental School, State University of Campinas, Av. Limeira, 901, Piracicaba, Brazil; 2 Faculty of Dentistry, Rio de Janeiro State University, Boulevard 28 de setembro, 157, Rio de Janeiro, Brazil; 3 Laboratory of Diphtheria and Corynebacteria of Clinical Relevance, Faculty of Medical Sciences, Rio de Janeiro State University, Boulevard 28 de setembro, 77, Rio de Janeiro, Brazil; 4 Computational and Systems Biology Laboratory, Oswaldo Cruz Institute, Oswaldo Cruz Foundation, Av. Brasil, 4365, Rio de Janeiro, Brazil

**Keywords:** Microbiome core, Saliva, MG-RAST, Amplicon sequencing, Shotgun metagenomics, eHOMD

## Abstract

**Background:**

Oral microbiota is considered as the second most complex in the human body and its dysbiosis can be responsible for oral diseases. Interactions between the microorganism communities and the host allow establishing the microbiological proles. Identifying the core microbiome is essential to predicting diseases and changes in environmental behavior from microorganisms.

**Methods:**

Projects containing the term “SALIVA”, deposited between 2014 and 2019 were recovered on the MG-RAST portal. Quality (*Failed*), taxonomic prediction (*Unknown* and *Predicted*), species richness (*Rarefaction*), and species diversity (*Alpha*) were analyzed according to sequencing approaches (Amplicon sequencing and Shotgun metagenomics). All data were checked for normality and homoscedasticity. Metagenomic projects were compared using the Mann–Whitney U test and Spearman's correlation. Microbiome cores were inferred by Principal Component Analysis. For all statistical tests, *p* < 0.05 was used.

**Results:**

The study was performed with 3 projects, involving 245 Amplicon and 164 Shotgun metagenome datasets. All comparisons of variables, according to the type of sequencing, showed significant differences, except for the *Predicted*. In Shotgun metagenomics datasets the highest correlation was between *Rarefaction* and *Failed* (r =  − 0.78) and the lowest between *Alpha* and *Unknown* (r =  − 0.12). In Amplicon sequencing datasets, the variables *Rarefaction* and *Unknown* (r = 0.63) had the highest correlation and the lowest was between *Alpha* and *Predicted* (r =  − 0.03). Shotgun metagenomics datasets showed a greater number of genera than Amplicon. *Propionibacterium*, *Lactobacillus*, and *Prevotella* were the most representative genera in Amplicon sequencing. In Shotgun metagenomics, the most representative genera were *Escherichia*, *Chitinophaga,* and *Acinetobacter*.

**Conclusions:**

Core of the salivary microbiome and genera diversity are dependent on the sequencing approaches. Available data suggest that Shotgun metagenomics and Amplicon sequencing have similar sensitivities to detect the taxonomic level investigated, although Shotgun metagenomics allows a deeper analysis of the microorganism diversity. Microbiome studies must consider characteristics and limitations of the sequencing approaches. Were identified 20 genera in the core of saliva microbiome, regardless of the health condition of the host. Some bacteria of the core need further study to better understand their role in the oral cavity.

**Supplementary Information:**

The online version contains supplementary material available at 10.1186/s12903-021-01719-5.

## Background

Metagenomic is a technique for accessing non-cultivable microorganisms DNA from environmental samples [1]. Since the development of New Generation Sequencing (NGS), this technique has been widely used in a number of scientific studies [2–4]. However, one of the challenges in metagenomics is to work with a large volume of data generated by sequencing and analysis. In bioinformatics, sequencing data must be deposited in a public database for wide access to be published in a scientific article. Therefore, since 2008 several specialized databases have allowed the deposit of raw and analyzed data from metagenomics projects [5, 6]. One of the pioneers in storage platforms for metagenomic data analysis is the public access portal MG-RAST [6].

The MG-RAST portal has deposited projects generated from different metagenomics approaches: Amplicon sequencing, Shotgun metagenomics and Metatranscriptomic. Amplicon sequencing (or metabarcoding) is done using the products of the polymerase chain reaction (PCR) that amplify the marker genes, such as 16S rRNA, 23S rRNA and 18S rRNA. Shotgun metagenomics has been used for total DNA sequencing from environmental samples while Metatranscriptomic has been used for sequencing all RNA extracted from investigated samples.

The oral microbiota refers to the collection of microorganisms that inhabit the oral cavity in different locations, such as the tongue, saliva and teeth [7]. Saliva has been shown to be a biological material capable of reflecting the dynamics of health conditions and metabolic, immunological or infectious diseases, reflecting dysbiosis of local and systemic origin, as observed in dental caries, periodontal diseases, diabetes, rheumatoid arthritis [8], cancer and, more recently, SARS-COV-2 [9, 10]. Obtaining salivary samples is simple, easy to perform, non-invasive, does not cause discomfort to the patient, is inexpensive, and representative of the oral environment. Saliva, like other biological fluids, contains DNA, RNA, proteins, and metabolic products, which are components of the host and its microbiota and their interactions. Despite the ease of its collection, knowledge of the behavior of the salivary microbiota and other constituents is still a challenge. Studies using sophisticated analysis such as the NGS, allow the investigation of differences in the bacterial profile in patients with oral diseases, such as caries and periodontitis, compared to healthy individuals [11].

The oral human microbiome, considered as the second most complex, is composed of more than 700 species [12] of which 54% are cultivable, 14% cultivable but unidentifiable, in addition to 32% of microorganisms unable to be cultured and identified [13].

Recent studies have proven the existence of an intrinsic relationship between the environment conditions and microbiome profiles [3, 13, 14]. Oral microbiota dysbiosis can be responsible for oral diseases, such as caries, plaque and periodontics [15–17]. Host-microbiological interactions allow establishing differences in microbiome profiles due to physiological and pathological conditions [18]. During the last decades, the development of methodologies and analyses for the identification and characterization of microbiomes has made it possible to predict diseases associated with changes in the environment and their reflexes in the microbiota, particularly those that share the same niche [19].

Description of microorganisms that share the same niche is called a core [19]. Identifying the core microbiome is essential to define how “healthy” this environment is [19]. The literature points out that the 10% prevalent microorganisms in the core must be considered dominant. On the other hand, the 65% less predominant should be considered rare [20].

Therefore, the relationship of this core with the environment, predicting diseases, and changes in environmental behavior from microorganisms not belonging to the core need further investigation [19].

## Main text

### Methods

This study aimed to investigate the core of the oral microbiome in saliva samples, regardless of host conditions by using the MG-RAST portal database.

Identifiers of metagenomes deposited between 2014 and 2019, containing as keyword the term "saliva" in the Material variable (material = ’SALIVA’) were selected for the study. A python in-house script was developed to extract, transform and load metadata from selected metagenomes to be filtered and analyzed. In order to assess the differences between sequencing approaches, only metagenomic projects that contained Amplicon sequencing and Shotgun metagenomics approaches were used in this study. In addition, only projects that had more than 3 metagenomes in each of the approaches were used due to statistical inferences.

Metagenomes were analyzed according to quality (*Failed*) and taxonomic prediction (*Unknown* and *Predicted*). In addition, metagenomes were analyzed according to species richness (*Rarefaction*) and species diversity (*Alpha*) (Table 1). Taxonomic data were recovered from the projects selected for the study of the core of the microbiome. Genus level was chosen for the analysis because Amplicon sequencing approach is more consistent for this taxonomic level [21].

**Table 1:** Variables recovered in MG-RAST

Variable	Description	MG-RAST name
*Failed*	Low quality sequences	QC failed
*Unknown*	Unrecognized sequence in public databases	QC unknown
*Predicted*	Sequence with inferred taxonomy	QC predicted
*Alpha*	Alpha diversity by Shannon index	ALPHA
*Rarefaction*	Max value of rarefaction curve	RAREFACTION
*Type*	Sequencing approaches	Sequence type
*Taxonomy*	Taxonomy at genus level	Taxonomy

The variables *Failed*, *Unknown*, *Predicted*, *Alpha*, and *Rarefaction* were used in the descriptive and correlation analyses. *Type* represents the sequencing approaches (Amplicon sequencing and Shotgun metagenomics). The *Taxonomy* variable contains the genera found in the samples that were used in the Principal Components Analysis

All data were checked for normality and homoscedasticity by Shapiro–Wilk test. Amplicon sequencing and Shotgun metagenomics data were compared using the Mann–Whitney U test. Associations among *Failed*, *Unknown*, *Predicted*, *Alpha*, and *Rarefaction* were performed by Spearman’s correlation. Microbiome cores were obtained and analyzed by Principal Component Analysis (PCA), to evaluate the most representative organisms of Amplicon sequencing and Shotgun metagenomics.

To allow a comparison between the results obtained in this study and the current literature, representative data of the oral microbiome from the expanded Human Oral Microbiome Database (eHOMD) [22] were used. eHOMD data were retrieved using the Taxon Table available at http://www.homd.org/?name=HOMD. After setting the Body Site filter field by checking only the Oral option, the text file was downloaded. Only the information in the Genus column was considered. Then the redundancies were removed and the remaining genera were considered the microbiome core of eHOMD. Microbiome cores obtained in the Amplicon sequencing and Shotgun metagenomics were compared with the microbiome core of the eHOMD by Venn diagram.

Statistical analysis was performed using the software R v3.6.1 [23]. The libraries *dplyr*, *ggplot2*, *reshape2*, and *data.table* were used in the descriptive analysis and correlation study. The *factoextra* library was used in the PCA. The value of *p* < 0.05 was used in all tests.

Python and R scripts are available on GitHub (https://github.com/rodrigojardim/mgrast-search).

In order to obtain more information about the selected projects, a search was carried out on PUBMED using the information of the principal investigator and the description of the project, both available on MG-RAST.

## Results

The survey in MG-RAST identified 621 metagenomes. Python script recovered 476 metagenomes, distributed in 12 sequencing projects, of which 332 metagenomes were Amplicon sequencing, 142 Shotgun metagenomics, and 2 Metatranscriptomes. The remaining 145 metagenomes had no metadata available and were discarded, as well as the recovered metatranscriptomes.

Projects that contained metagenomes from both sequencing approaches were selected. In addition, 1 project that had only 1 metagenome for each approach was discarded. In this way, 3 projects with 245 Amplicon sequencing metagenomes and 164 Shotgun metagenomics metagenomes were used in this study (Table 2).

**Table 2:** Number of metagenomes by project and sequencing approaches

Project	Approaches	Number of metagenomes
mgp3474	Amplicon	95
mgp4843	Amplicon	97
mgp7236	Amplicon	53
mgp3474	Shotgun	8
mgp4843	Shotgun	73
mgp7236	Shotgun	83

**Fig. 1:**
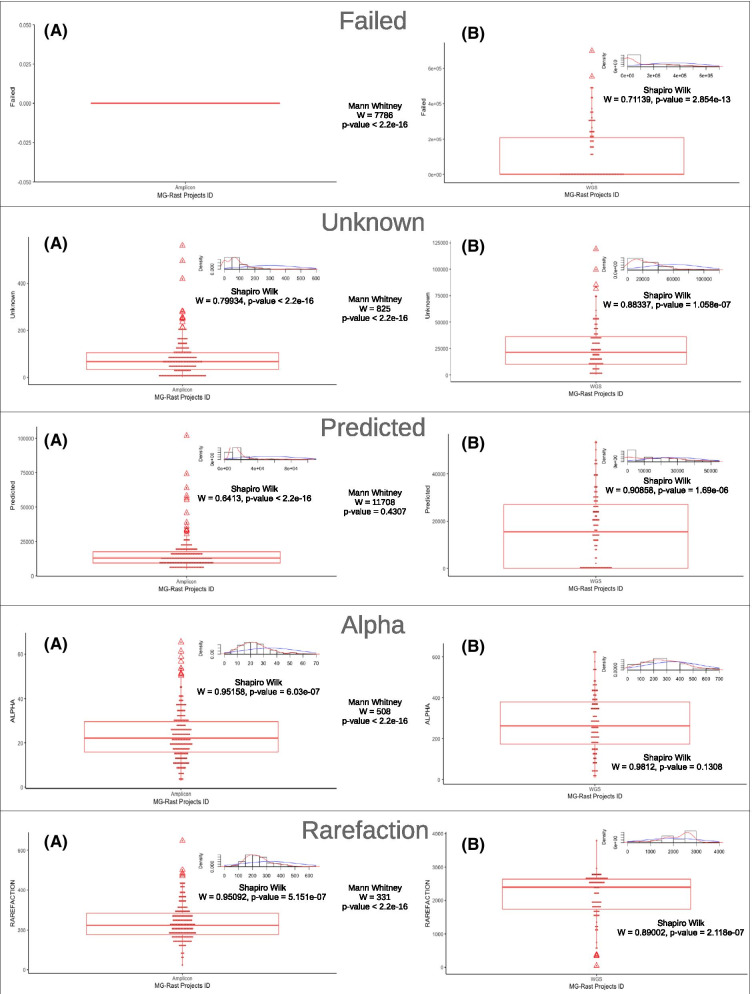
Descriptive analysis. Boxplot shows the data distribution of *Failed*, *Unknown*, *Predicted*, *Alpha* and *Rarefaction* variables, comparing (**a**) Amplicon sequencing and (**b**) Shotgun metagenomics approaches. Normality analysis was performed using the Shapiro Wilk test and the Mann–Whitney U test evaluated the differences of the variables between the sequencing approaches. All variables showed a significant difference (*p* < 0*.*05) between Amplicon sequencing and Shotgun metagenomics, except *Predicted*. Note that *Failed* did not present any data in the Amplicon sequencing

Figure 1 shows the results of the descriptive analysis of the numerical variables. For the Amplicon sequencing datasets there was no sequence with quality problems. All variables of both approaches had a non-Gaussian distribution, with the exception of the *Alpha* in Shotgun metagenomics. All comparisons of variables, according to the sequencing approaches, showed significant differences (*p* < 0.05), except for the *Predicted* (*p* = 0.4307).

**Fig. 2:**
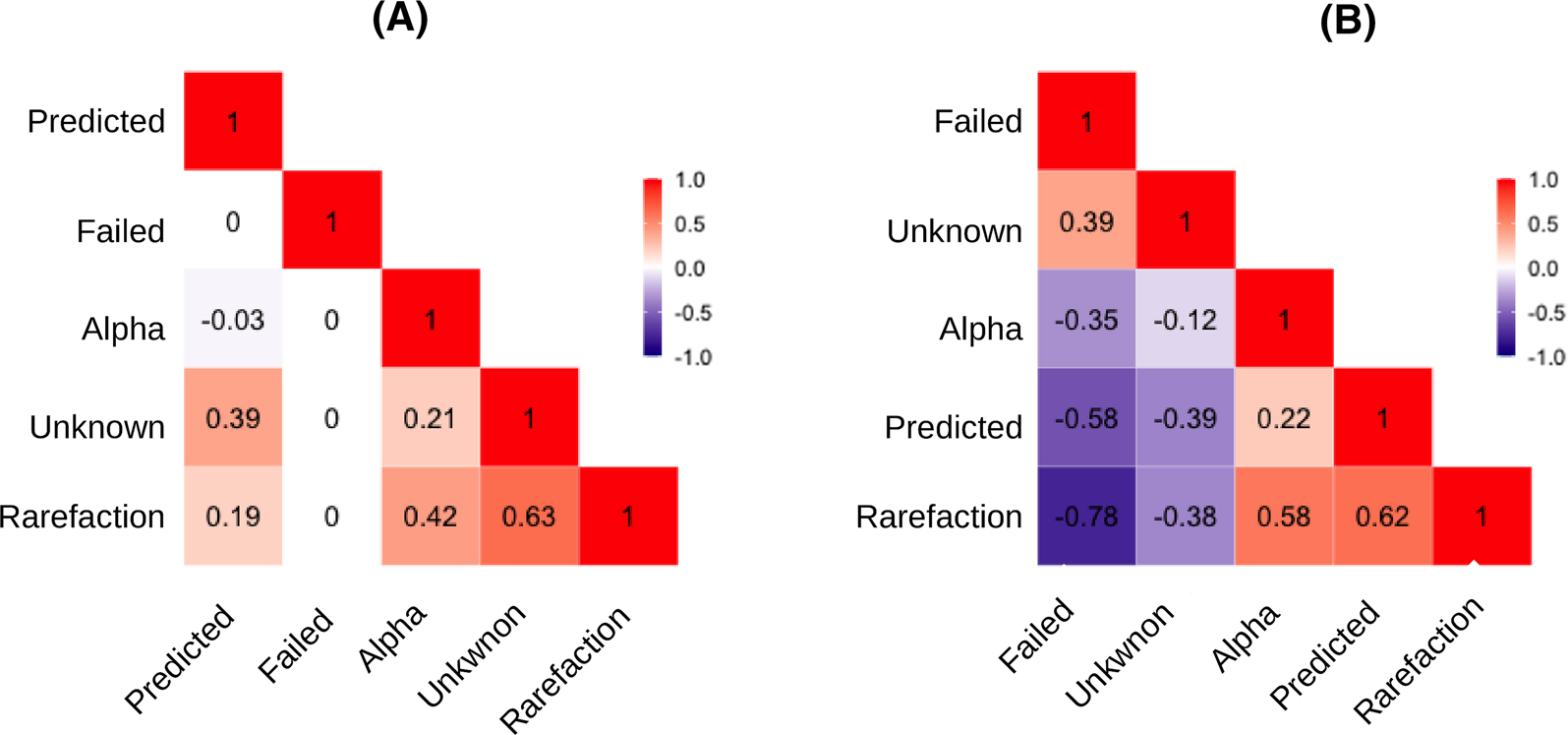
Correlation analysis. The Spearman’s test evaluated the associations between *Failed*, *Unknown*, *Predicted*, *Alpha*, and *Rarefaction* variables of **a** Amplicon sequencing and **b** Shotgun metagenomics. All associations were positive in Amplicon sequencing. In the Shotgun metagenomics, *Failed* and *Unknown* were negatively associated with *Predicted*, *Alpha*, and *Rarefaction*

Correlation study of the five variables showed divergences between sequencing approaches (Fig. 2). Most of the correlations of the variables in Amplicon sequencing datasets were positive, in contrast to what was observed in Shotgun metagenomics. In Shotgun datasets, the highest correlation was between *Rarefaction* and *Failed* (*r* =  − 0.78) and the lowest between *Alpha* and *Unknown* (*r* =  − 0.12). In Amplicon datasets, *Rarefaction* and *Unknown* (*r* = 0.63) had the highest correlation and the lowest was between *Alpha* and *Predicted* (*r* =  − 0.03).

**Fig. 3:**
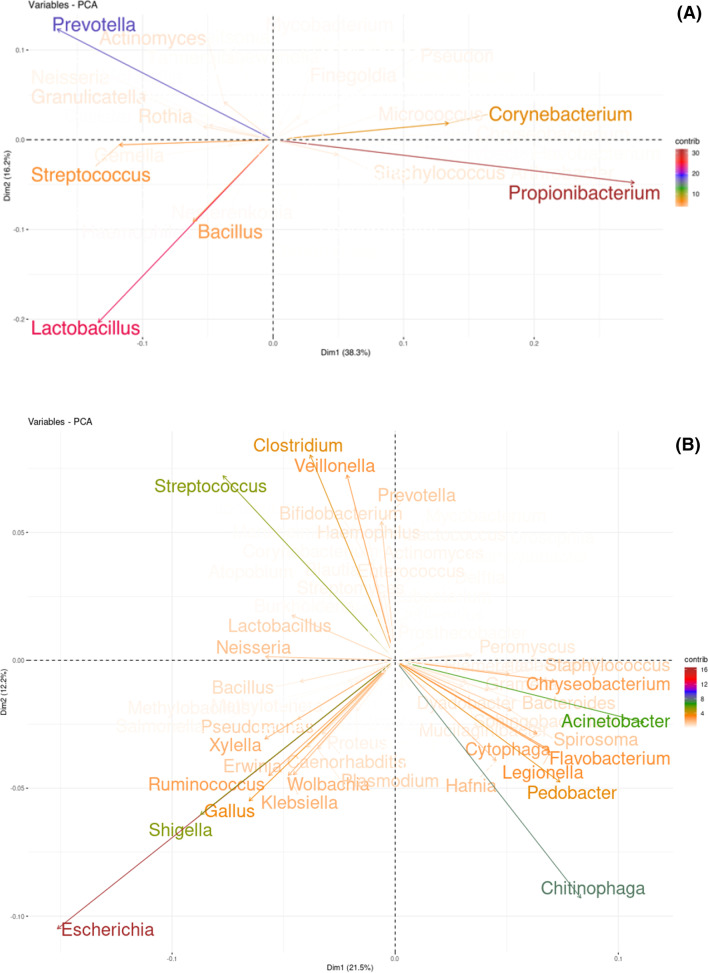
Principal Components Analysis. Bi-plot showing the representativeness of the genera present in the core of both approaches: **a** Amplicon sequencing and **b** Shotgun metagenomics. Graphs show the dimensions with greatest variance.

**Fig. 4:**
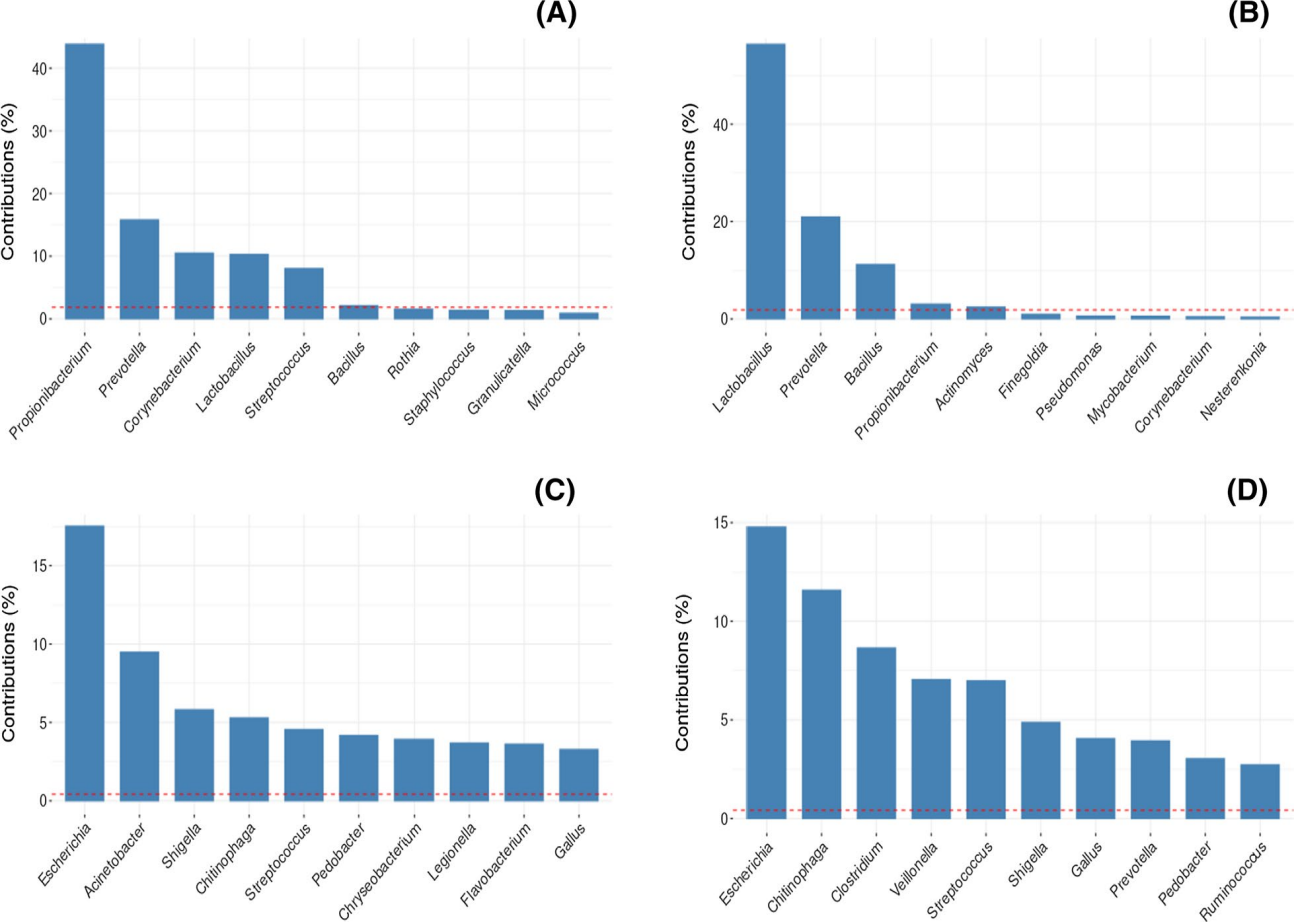
Top 10 bacteria in the human saliva microbiome. Contribution of the main genera in dimensions 1 and 2 of the Principal Component Analysis (PCA) of the Amplicon sequencing and Shotgun metagenomics approaches. Amplicon sequencing: **a** dimension 1 and **b** dimension 2. Shotgun metagenomics: **c** dimension 1 and **d** dimension 2

In the PCA, microbiome cores with different genera were found between Amplicon sequencing and Shotgun metagenomics. Number of genera obtained by the Shotgun dataset was greater than that observed in the Amplicon dataset. The top 10 genera showed that only *Prevotella* and *Streptococcus* are representative in cores of both approaches. *Propionibacterium*, *Lactobacillus* and *Prevotella* were the most representative genera in Amplicon sequencing. On the other hand, *Escherichia*, *Chitinophaga*, and *Acinetobacter* (Figs. 3 and 4) were the most representative genera in Shotgun metagenomics. The genera present in the microbiome core of both Shotgun and Amplicon are listed in Additional file 1: S1.

**Fig. 5:**
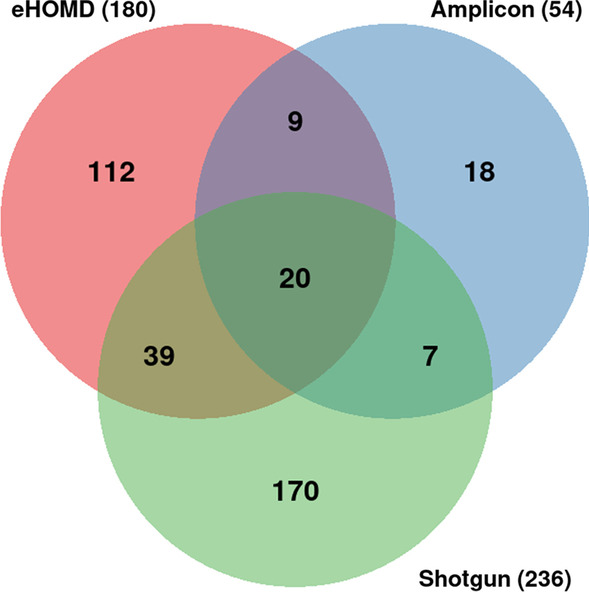
Core of saliva microbiomes. Venn diagram showing microbiome cores shared among Amplicon sequencing, Shotgun metagenomics, and eHOMD

By comparing Shotgun metagenomics, Amplicon sequencing and eHOMD datasets a common or shared microbiome core containing 20 genera was found (Table 3). eHOMD and Shotgun metagenomics datasets share a microbiome core composed of 39 genera, and eHOMD and Amplicon sequencing share a core with only 9 genera. Shotgun and Amplicon datasets share a core containing 7 genera (Fig. 5). The complete genera names sharing the different microbiome cores is in Additional file 2: S2.

**Table 3:** Bacteria genera of human saliva microbiome core and their relationship with oral diseases

Gram staining	Genus	Relation with oral disease
Gram-negative	*Acinetobacter*	Oral squamous cell carcinoma [24]
	*Delftia*	Geographic tongue [25]
	*Dialister*	Sjøgren’s syndrome [26]
	*Enterobacter*	Denture stomatitis [27]
	*Haemophilus*	Squamous cell carcinoma [28]
	*Moraxella*	Peri-implantitis [29]
	*Neisseria*	Healthy periodontal conditions [30]
	*Prevotella*	Periodontal disease [30]
	*Pseudomonas*	Oral cancer [31]
	*Ralstonia*	Periodontitis [32]
	*Sphingomonas*	Recurrent aphthous ulcer [33]
Gram-positive	*Actinomyces*	Dental caries [34]
	*Bacillus*	Dental caries [35]
	*Corynebacterium*	Biofilm formation [36]
	*Lactobacillus*	Dental caries [34]
	*Micrococcus*	Lysozyme activity [37]
	*Mycobacterium*	Dental infections [38]
	*Rothia*	Dental caries [39]
	*Staphylococcus*	Acute sialadenitis [40]
	*Streptococcus*	Dental caries [34]

Search in PUBMED identified 12 articles published between 2011 and 2015, with the PMIDs: 25994215, 25861745, 25020228, 24981669, 24903519, 24846382, 24646696, 23598790, 22583485, 2254158393, 21149389, and 205478343.

## Discussion

Bacteria are highly prevalent microorganisms in the microbiota and play an important role in oral homeostasis [41]. The abundance of some bacteria may indicate dysbiosis of the oral microbiome [25, 35]. Identification of the core is inferred from the study of different microbiomes and allows to reveal the conditions of the hosts according to the presence/absence or predominance of some species over others. However, there is a set of bacteria that, regardless of the condition of the host, can be part of the core of all these microbiomes. Similar to the concept of housekeeping genes, which conceptualize the genes essential to the life of an organism, a set of bacteria from the oral microbiome, regardless of the health status of the host, can be inferred as that essential to the symbiosis between the microorganisms of the oral cavity.

In this study, 20 genera of bacteria were found in more than 450 metagenomes (Fig. 5) deposited in public databases and which, regardless of the health condition of the host, are present in the core of the oral microbiome.

In both Amplicon sequencing and Shotgun metagenomics cores, genera of bacteria already associated with caries and periodontal diseases were found, such as *Streptococcus*, *Lactobacillus* and *Prevotella* [10, 42].

The saliva of individuals with high caries experience is associated with a high salivary abundance of *Streptococcus* and countless species of *Lactobacillus* in addition to other bacteria capable of degrading sugars and forming extracellular polysaccharides [18]. Samples from healthy individuals with low caries experience were associated with a greater abundance of the genera *Neisseria*, *Haemophilus*, and *Fusobacterium*, of which most species of this genera only ferment sugar slightly [43]. According to Tanner et al. [18], the composition of saliva in the oral cavity is one of the main risk factors associated with caries. Biofilm dysbiosis results in an increase in acidogenic and aciduric species, capable of modulating the core components in the biofilm. While in cases of gingivitis, the increase in the amount of plaque around the gingival margin induces the inflammatory response in the host, leading to increased levels of anaerobic bacteria, including Gram-negative proteolytic species, especially those belonging to the *Prevotella*, *Porphyromonas*, *Tannerella*, *Fusobacterium* and *Treponema* genera [44].

Identification of *Corynebacterium*, *Escherichia*, *Pseudomonas* and *Shigella* suggests that genera with pathogenic potential may also be part of the core of the oral microbiome obtained by salivary samples. *Chitinophaga* was a recently described taxon and was observed only in Shotgun metagenomics. This genus was highly representative, as well as *Escherichia*, *Acinetobacter*, *Streptococcus*, and *Shigella* (Fig. 3). The pathogenic potential of the genus *Chitinophaga* has already been reported [45, 46]. However, its role in the oral microbiome is still unknown.

Amplicon sequencing metadata analysis showed inconsistent behavior (Fig. 2). Only *Rarefaction* and *Unknown* had correlation greater than 0.50 (*r* = 0.63). The expected behavior was of inverse correlation. The greatest number of non-inferred sequences determines the smallest number of potentially discovered organisms.

On the other hand, Shotgun metagenomics presented results as expected, which can be exemplified by inverse correlation between *Failure* and *Rarefaction*. The largest number of sequences with quality failure determines the smallest number of sequences to be inferred, which affects the rarefaction curve. This was exactly the behavior observed in Shotgun metagenomics for these variables (*r* =  − 0.78).

These behaviors in different approaches certainly influenced the comparative study between them (Fig. 1). Furthermore, it was observed in the Amplicon sequencing dataset that all projects did not present sequences with quality failures (*Failed* = 0). This result was unexpected. Even Amplicon sequencing can have quality failures in the sequencing process. However, the *Predicted* was the only one that did not show difference between the approaches, showing results similar to those observed by [47], who investigated the microbial composition of the human intestine.

Results obtained by Shotgun metagenomics allowed a more complex characterization of the microbiome, with the identification of greater diversity and at the taxonomic level of species, when compared to Amplicon sequencing which uses regions of the gene with variability to identify down to the genus level [48].

According to the literature [47, 49], the PCA identified a greater number of representative genera in the Shotgun dataset than in the Amplicon dataset (Fig. 3). The differences between them may explain the findings. In Shotgun metagenomics, the DNA of all the organisms in the sample is extracted and sequenced directly. On the other hand, in Amplicon sequencing, only the DNA fragments that were aligned to the primer will be sequenced. The choice of primer seems to be a crucial factor to avoid bias in taxonomic analysis [50].

The specificity of primers may restrict the set of microorganisms found in studies of Amplicon sequencing. Thus, the choice of the sequencing method as well as the selection of primers are important characteristics to be considered in the analysis of microbiome studies [51].

Microbiome studies comparing the two sequencing methods for the same samples suggest that their results might be comparable. In this study, we observed that the data produced by Shotgun metagenomics of salivary samples available on the MG-RAST platform can provide the identification of a greater number of genera, evidencing the complexity of the oral microbiome, either by the diversity of genera or by the role they may play in the salivary microbiome [50].

These results should be interpreted with caution, since only the presence of the genera does not determine the condition of the host. Other characteristics such as abundance and interaction between genera have a relevant role in the association of the microbiota with the condition of the host [12].

Metagenomics projects deposited in public databases such as eHOMD and MG-RAST do not always provide information on the health conditions of the host, DNA/RNA extraction techniques or other information that might infer microbiome-host relationships.

Studies identified using the information of the principal investigator suggest that they correspond to the data obtained in the MG-RAST. However, it is not possible to specify whether such articles refer to data investigated in this study. According to the MG-RAST pipeline guideline (https://help.mg-rast.org/user_manual.html), it is not possible to carry out analyses of eukaryotes or viruses, which suggests that the DNA/RNA extraction method of the selected projects allows inferring the bacterial microbiota.

## Conclusions

This study demonstrated that in the microbiota representative of human saliva, genera of pathogenic bacteria observed in oral diseases were identified, but not limited to them.

Core of the salivary microbiome and genera diversity are dependent on the sequencing approaches. Available data suggest that Shotgun metagenomics and Amplicon sequencing have similar sensitivities to detect the taxonomic level investigated, although Shotgun metagenomics allows a deeper analysis of the microorganism diversity.

The choice of metagenomics approaches must consider their characteristics and limitations. Shotgun metagenomics sequencing can provide a great contribution to the knowledge of the composition of the salivary microbiota, identification of markers for diagnosis and identification of profiles capable of defining health or disease conditions. On the other hand, Amplicon sequencing can be an efficient and low-cost choice in studies in which the microorganism of interest is already known. It can also be used for further verification of results obtained by Shotgun metagenomics.

## Supplementary Information

**Additional file 1**: S1 - Microbiome core: Shotgun and Amplicon. Text file with a list of genera belongs to the microbiome core of Shotgun metagenomics and Amplicon sequencing.

**Additional file 2**: S2 - Microbiome core among Shotgun, Amplicon, and eHOMD. Text file with a list of genera belongs to the microbiome core among Shotgun metagenomics, Amplicon sequencing, and eHOMD.

## Data Availability

Data was deposited in the Mendeley Data (https://doi.org/10.17632/vvwmvvxxs4.1). Scripts are available at https://github.com/rodrigojardim/mgrast-search.

## Abbreviations

NGS: New generation sequencing.
PCR: Polymerase chain reaction.
PCA: Principal components analysis.
eHOMD: Expanded Human Oral Microbiome Database
