# Supplementary material for: Core of the saliva microbiome: an analysis of the MG-RAST data
Source: BMC Oral Health. 2021 Jul 16;21:351. doi: 10.1186/s12903-021-01719-5 (PMC8283749; doi:10.1186/s12903-021-01719-5)
Supplement: Supplementary file 1 — Additional file 1: S1 - Microbiome core: Shotgun and Amplicon. Text file with a list of genera belongs to the microbiome core of Shotgun metagenomics and Amplicon sequencing. [file 12903_2021_1719_MOESM1_ESM.pdf]

Core of the saliva microbiome: an analysis of the MG-RAST data

Simone G Oliveira[1,2] , Rafaela R. Nishiyama[1], Claudio A.C. Trigo[1], Ana Luiza de M Guaraldi[3], Alberto M R Dávila[4], Rodrigo Jardim[4*] and Flavio H.B. Aguiar[1]

Material Supplementary

S1 - Microbiome core: Shotgun and Amplicon

Amplicon sequencing

Acinetobacter
Actinomyces
Aeromonas
Alicyclobacillus
Amycolatopsis
Aquabacterium
Arthrobacter
Bacillus
Brachybacterium
Candidatus Glomeribacter
Carnobacterium
Cellulomonas
Chryseobacterium
Comamonas
Corynebacterium
Cupriavidus
Delftia
Dialister
Empedobacter
Enterobacter
Finegoldia
Flavobacterium
Gemella
Granulicatella
Haemophilus
Kocuria
Lactobacillus
Leifsonia
Massilia
Methylobacterium
Micrococcus
Mitsuaria
Mobiluncus
Moraxella
Mycobacterium
Neisseria
Nesterenkonia
Nocardioides
Oerskovia
Okibacterium
Prevotella
Propionibacterium
Pseudomonas
Psychrobacter
Ralstonia
Rhizobium
Rothia
Shewanella
Sphingobium
Sphingomonas
Staphylococcus
Streptococcus
Tannerella
Tetrasphaera

Shotgun metagenomics

Achromobacter
Acidithiobacillus
Acidobacterium
Acidovorax
Acinetobacter
Actinomyces
Aedes
Aggregatibacter
Agrobacterium
Akkermansia
Albidiferax
Algoriphagus
Alkalilimnicola
Anabaena
Anaeromyxobacter
Anaerostipes
Aspergillus
Atopobium
Avibacterium
Bacillus
Bacteroides
Basfia
Bifidobacterium
Blastopirellula
Blautia
Bordetella
Bpp-1-like viruses
Bradypus
Bradyrhizobium
Branchiostoma
Brevibacillus
Brevibacterium
Burkholderia
Caenorhabditis
Campylobacter
Candidatus Azobacteroides
Candidatus Koribacter
Candidatus Pelagibacter
Candidatus Solibacter
Capnocytophaga
Capra
Capsaspora
Caulobacter
Cellvibrio
Chelativorans
Chitinophaga
Chlorobaculum
Chlorobium
Chryseobacterium
Chthoniobacter
Ciona

Citrobacter
Clostridium
Collinsella
Conexibacter
Corynebacterium
Coxiella
Croceibacter
Cupiennius
Cyanothece
Cytophaga
Danio
Dechloromonas
Dehalococcoides
Delftia
Desulfobacterium
Desulfotomaculum
Dialister
Dorea
Drosophila
Dyadobacter
Eikenella
Enhydrobacter
Enterobacter
Enterococcus
Equus
Erwinia
Escherichia
Ethanoligenens
Eubacterium
Faecalibacterium
Flavobacterium
Fluoribacter
Frankia
Fusobacterium
Gallionella
Gallus
Geobacter
Glaciecola
Gloeobacter
Gluconobacter
Gordonibacter
Gramella
Haemophilus
Hafnia
Histophilus
Homo
Idiomarina
Ilyobacter
Inovirus
Janthinobacterium
Jonesia
Kingella
Klebsiella
Kluyvera
Kluyveromyces
Kordia
Ktedonobacter
Lactobacillus
Lactococcus
Lambda-like viruses
Laribacter
Leadbetterella
Leavenworthia
Leeuwenhoekiella
Legionella
Leifsonia
Lentisphaera
Leptospira
Leuconostoc
Listeria
Lodderomyces
Lutiella
Magnetospirillum
Malassezia
Mannheimia
Maribacter
Marinobacter
Marivirga
Megasphaera
Methanosphaera
Methylibium
Methylobacillus
Methylotenera
Methylovorus
Micrococcus
Microcoleus
Microscilla
Moraxella
Mu-like viruses
Mucilaginibacter
Mycobacterium
Mycoplasma
Myroides
Myxococcus
Neisseria
Nitratifractor
Nitrobacter
Nitrosococcus
Nitrospira
Nocardia
Nodularia
Nostoc
Octadecabacter
Odocoileus
Oribacterium
Oryctolagus
P2-like viruses
Paenibacillus
Pantoea
Parabacteroides
Paracoccus
Pasteurella
Pedobacter
Peromyscus
Pirellula
Piromyces
Planctomyces
Plasmodium
Polaribacter
Polaromonas
Populus
Prevotella
Prosthecobacter
Proteus
Pseudomonas
Pseudovibrio
Psychrobacter
Ralstonia
Rhizobium
Rhodobacter
Rhodococcus
Rhodopirellula
Rhodopseudomonas
Rhodospirillum
Rhodothermus
Ricinus
Riemerella
Roseburia
Roseobacter
Rothia
Ruegeria
Ruminococcus
Saccharophagus
Salinibacter
Salinispora
Salmonella
Schistosoma
Sebaldella
Selenomonas
Serratia
Shewanella
Shigella
Sideroxydans
Sinorhizobium
Solobacterium
Sphingobacterium

Sphingobium
Sphingomonas
Spirosoma
Stackebrandtia
Staphylococcus
Stigmatella
Streptococcus
Streptomyces
Sulfitobacter
Synechococcus
Synechocystis
T7-like viruses
Terrimonas
Tetraodon
Thauera
Thermobaculum
Tityus
Treponema
Trypanosoma
Veillonella
Verminephrobacter
Verrucomicrobium
Wolbachia
Xanthomonas
Xenopus
Xylella
Yersinia
Zingiber
Zunongwangia
