# Supplementary material for: Core of the saliva microbiome: an analysis of the MG-RAST data
Source: BMC Oral Health. 2021 Jul 16;21:351. doi: 10.1186/s12903-021-01719-5 (PMC8283749; doi:10.1186/s12903-021-01719-5)
Supplement: Supplementary file 2 — Additional file 2: S2 - Microbiome core among Shotgun, Amplicon, and eHOMD. Text file with a list of genera belongs to the microbiome core among Shotgun metagenomics, Amplicon sequencing, and eHOMD. [file 12903_2021_1719_MOESM2_ESM.pdf]

Core of the saliva microbiome: an analysis of the MG-RAST data

Simone G Oliveira[1,2] , Rafaela R. Nishiyama[1], Claudio A.C. Trigo[1], Ana Luiza de M Guaraldi[3], Alberto M R Dávila[4], Rodrigo Jardim[4*] and Flavio H.B. Aguiar[1]

Material Supplementary

S2 - Microbiome core among Shotgun, Amplicon and HOMD

<u>Amplicon x Shotgun</u>

Acinetobacter	Haemophilus	Psychrobacter
Actinomyces	Lactobacillus	Ralstonia
Bacillus	Leifsonia	Rhizobium
Chryseobacterium	Micrococcus	Rothia
Corynebacterium	Moraxella	Shewanella
Delftia	Mycobacterium	Sphingobium
Dialister	Neisseria	Sphingomonas
Enterobacter	Prevotella	Staphylococcus
Flavobacterium	Pseudomonas	Streptococcus

<u>Amplicon x HOMD</u>

Acinetobacter	Gemella	Prevotella
Actinomyces	Granulicatella	Propionibacterium
Bacillus	Haemophilus	Pseudomonas
Comamonas	Kocuria	Ralstonia
Corynebacterium	Lactobacillus	Rothia
Cupriavidus	Micrococcus	Sphingomonas
Delftia	Mobiluncus	Staphylococcus
Dialister	Moraxella	Streptococcus
Enterobacter	Mycobacterium	Tannerella
Finegoldia	Neisseria	

<u>Shotgun x HOMD</u>

Achromobacter	Burkholderia	Fusobacterium
Acidovorax	Campylobacter	Haemophilus
Acinetobacter	Capnocytophaga	Kingella
Actinomyces	Caulobacter	Klebsiella
Aggregatibacter	Corynebacterium	Kluyvera
Agrobacterium	Delftia	Lactobacillus
Atopobium	Dialister	Lactococcus
Bacillus	Eikenella	Listeria
Bacteroides	Enhydrobacter	Megasphaera
Bifidobacterium	Enterobacter	Micrococcus
Bordetella	Enterococcus	Moraxella
Bradyrhizobium	Escherichia	Mycobacterium
Brevibacterium	Eubacterium	Mycoplasma

Neisseria
Oribacterium
Paenibacillus
Paracoccus
Pedobacter
Prevotella
Proteus
Pseudomonas
Ralstonia
Rhodobacter
Rothia
Selenomonas
Serratia
Solobacterium
Sphingomonas
Staphylococcus
Streptococcus
Treponema
Veillonella
Yersinia
